# Microwave-Assisted Coal-Derived Few-Layer Graphene as an Anode Material for Lithium-Ion Batteries

**DOI:** 10.3390/ma14216468

**Published:** 2021-10-28

**Authors:** Faridul Islam, Jialong Wang, Arash Tahmasebi, Rou Wang, Behdad Moghtaderi, Jianglong Yu

**Affiliations:** 1 Chemical Engineering, School of Engineering, The University of Newcastle, Callaghan, NSW 2308, Australia; faridul.islam@uon.edu.au (F.I.); arash.tahmasebi@newcastle.edu.au (A.T.); rou.wang@uon.edu.au (R.W.); behdad.moghtaderi@newcastle.edu.au (B.M.); 2 School of Environmental and Life Sciences, The University of Newcastle, Callaghan, NSW 2308, Australia; jialong.wang@uon.edu.au; 3 Monash Research Institute of Science and Technology (Suzhou Industrial Park), Southeast University—Monash University Joint Graduate School, Suzhou 215000, China

**Keywords:** few-layer graphene, coal, catalytic graphitization, lithium-ion batteries, microwave

## Abstract

A few-layer graphene (FLG) composite material was synthesized using a rich reservoir and low-cost coal under the microwave-assisted catalytic graphitization process. X-ray diffraction, Raman spectroscopy, transmission electron microscopy, and X-ray photoelectron spectroscopy were used to evaluate the properties of the FLG sample. A well-developed microstructure and higher graphitization degree were achieved under microwave heating at 1300 °C using the S5% dual (Fe-Ni) catalyst for 20 min. In addition, the synthesized FLG sample encompassed the Raman spectrum 2D band at 2700 cm^−1^, which showed the existence of a few-layer graphene structure. The high-resolution TEM (transmission electron microscopy) image investigation of the S5% Fe-Ni sample confirmed that the fabricated FLG material consisted of two to seven graphitic layers, promoting the fast lithium-ion diffusion into the inner surface. The S5% Fe-Ni composite material delivered a high reversible capacity of 287.91 mAhg^−1^ at 0.1 C with a higher Coulombic efficiency of 99.9%. In contrast, the single catalyst of S10% Fe contained a reversible capacity of 260.13 mAhg^−1^ at 0.1 C with 97.96% Coulombic efficiency. Furthermore, the dual catalyst-loaded FLG sample demonstrated a high capacity—up to 95% of the initial reversible capacity retention—after 100 cycles. This study revealed the potential feasibility of producing FLG materials from bituminous coal used in a broad range as anode materials for lithium-ion batteries (LIBs).

## 1. Introduction

Lithium-ion batteries (LIBs) are remarkable in many aspects owing to their high energy density, long cycling life, and low pollution levels [1,2,3]. The fast-growing global demand for portable electronic devices and electric and hybrid automobiles has led to much-advanced research on electrode materials [4]. Recent research has focused on improving the electrochemical performances of LIBs in such areas as stability, cycling capacity, and cost-effectiveness by looking at employing different kinds of carbon materials as anode materials. Carbon composite materials such as graphene, boron-doped carbon foam, artificial graphite scrap, and anthracite-based graphite have been used as anode materials to enhance electrochemical performance. In addition, graphene has an outstanding rate capability for battery performance due to its higher conductivity, long chemical stability, and ample storage capacity [5,6]. Furthermore, it has been found that synthetic graphite has excellent cycling and rate capacities for use as an anode material in LIBs [7]. In addition, the biomass reed flowers-derived bimodal porous carbon (BPC) was synthesized through sulfur mixing, providing a high Li-S battery performance and increasing the higher surface area [8]. According to the researcher, the FLG adhesion strength between the current collector and the active materials contributes to making a high-density electrical path and a high mechanical electrode strength within the electrode. These facilities are highly effective in functional materials volume change during the charge/discharge process and smooth the electron transfer. As a result, the FLG composite materials deliver a high power density and long-term energy storage capacity [9]. In general, a range of coals and petroleum cokes have been used as precursors to make the composite materials for anodes, and they require a high temperature (3000 °C) for graphitization [10,11]. However, the high production costs and complex procedures have affected their large-scale industrial applications.

Coal is a naturally rich carbon source that has been used as a precursor in the development of a range of carbon material products, including nanotubes [12], fullerene [13], and graphene nanosheets [14]. Several hydroaromatic and aromatic domains link short-chain aliphatic and ether groups in coal [15]. These linking groups detach at high pyrolysis temperatures, which changes the amorphous order into a graphitic structure [15]. Carbon materials such as bituminous coal have been pyrolyzed at a high temperature of 2800 °C [16]. In addition, anthracite coal has been graphitized at a range of high temperatures to measure its performance as an anode material in LIBs applications [17]. However, the cost of using carbonaceous materials for synthetic graphite production is higher due to the amount of time and energy consumed and the high processing temperatures required.

Microwaves (MW) have been used as a substitute energy source in several sectors, such as catalytic graphitization [18] and environmental remediation [19], due to several benefits: for instance, its volumetric heating capacity, shorter processing time, and lower energy consumption [20,21]. It has also been used to prepare graphene composite materials by transferring the MW energy into heat energy, where graphite oxide has been used as a carbon source [22]. In addition, the microwave has assisted in preparing the less oxygen-containing graphene from graphite oxide and exfoliating the high surface graphene due to its fast heating mode. Moreover, a solvent-free, biomass-derived FLG was synthesized at 1000 °C for 3 h under a catalytic (FeCl_3_·6H_2_O) carbothermal graphitization reaction. This few-layer graphene provided relatively high Hall mobility and electrical conductivity [23]. Furthermore, the chemical vapor deposition (CVD) method is commonly used as one of the most active procedures for synthesizing high-quality graphene [24]. Moreover, our previous study found that the microwave set temperature and optimum catalyst percentage played a crucial role in making the FLG composite material. At fixed microwave temperate (1300 °C for 20 min), graphitic layer formation through the dissolution and precipitation on the amorphous carbon matrix at the eutectic point of the catalyst and carbon made a good synchronization. The metal-supported FLG composite materials were synthesized at 1300 °C for 20 min using a single catalyst (S10% Fe) under microwave graphitization, making a unique morphology and a well-developed mesopore structure with three to six layers of graphene nanosheets [25].

Transition metals such as Fe, Ni, and Co are frequently used as catalysts to reduce the treatment temperatures and time required to produce composite materials with nanostructures [26,27]. Moreover, porous carbons have been prepared at low temperatures using a Ni catalyst, and Fe has also assisted in preparing composite materials with nanostructures [28,29]. As a result, the carbon order has changed from Fe^3+^ to Fe_3_O_4_, Fe_3_C, or metallic Fe due to the reduction and carbonization reaction during the graphitization period. Fe^3+^ has been used as a catalyst to produce the honeycomb carbon order reorganization on the surfaces of carbons during pyrolysis [30,31]. According to Arrigo et al., at 573 K below temperature, the Fe atoms are fast diffused at the edges of the graphene layers. However, at higher 873 K temperature, the Fe atoms formed highly stable three or four-coordinated atomic species together with dinuclear complex Fe species closer to the graphene layer edge. This diffusion has occurred from one site to the other very fast. As a result, the Fe agglomerated. In addition, the C-C bonds are included at the edges of the site of the graphene layer at a higher temperature by the metal Fe atoms [32]. Wang et al. used anthracite coal as a precursor to prepare synthetic graphite using several catalysts, including boric acid (H_3_BO_3_), lanthanum oxide (La_2_O_3_), cerium oxide (CeO_2_), and praseodymium oxide (Pr_6_O_11_), for LIB applications [33]. These catalysts also assisted in increasing the regular graphitic nanostructures. According to the Fe-C and Ni-C phase diagrams, the catalysts acted as nuclei to make the graphitic carbon layer through dissolution and precipitation on the amorphous carbon matrix at the supersaturation point of the catalyst and carbon [34,35]. Nevertheless, a stable and well-controlled graphitic structure with a cost-effective and straightforward process for the large-scale production of FLG for anode materials in LIBs is still required in the commercial arena.

This study prepared the FLG composite material from bituminous coal using iron (III) nitrate nonahydrate and nickel (II) nitrate hexahydrate as catalysts under microwave heating temperature. This work significantly decreased the processing time and the fabrication costs of making the FLG. Furthermore, the morphology and performances of the FLG were measured using several analytical techniques.

## 2. Materials and Methods

### 2.1. Materials and Sample Preparation

Australian bituminous coal was used to produce FLG composite materials. The coal was processed (particle size of <63 μm) and then steam gasified to raise the surface areas of the carbon materials, which was described in detail in our previous study [25]. Then, the Fe(NO_3_)_3_·9H_2_O and Ni(NO_3_)_2_·6H_2_O, with several catalyst loading weight percentages, were loaded as catalysts to impregnate the coal particles. The coal and double catalyst samples were mixed overnight (for 12 h) by a magnetic stirrer; then, 0.1 M of NH_4_OH solution was added for 30 min, which was followed by washing with de-ionized water. Lastly, the solid residue was collected using filtration and dried using an oven at 110 °C. The steam-activated Fe and Ni catalyst-loaded samples were denoted as S2% Fe-Ni, S5% Fe-Ni, S10% Fe-Ni, and S20% Fe-Ni.

### 2.2. Synthesis of the FLG Composite Materials

The FLG samples were synthesized using a microwave oven and a custom-designed quartz reactor. A high-temperature B-type thermocouple and N_2_ gas were used in this synthesis process, as explained in our earlier paper [25]. All of the experiments used a 4 g sample heated at a temperature of 1300 °C for 20 min in microwave radiation with an N2 gas flow rate of 250 mL/min. The microwave oven had an automated system for controlling the heating and cooling rate and power systems. The samples were collected from the microwave oven after the display showed that the experiment finished. Furthermore, the single S10% Fe catalyst samples were synthesized using the same method. The produced composite materials were used to compare their electrochemical performances with the dual catalyst-loaded FLG materials reported in the literature [25].

### 2.3. Characterization of the FLG Composite Materials

X-ray diffraction (Bruker, Germany) analysis was performed to examine the crystalline structures of the samples in a scanning range from 10° to 80°. The interlayer spacings (d_002_), crystal sizes (La), and crystal heights (Lc) of the samples were calculated using the Debye–Scherer equation, and the g factor values were measured using the Marie and Meiring equation [36]. Raman spectroscopy (Horiba XploRA PLUS) evaluated the crystal structural order in the wavenumber range of 500–3000 cm^−1^ using a He-Ne laser at an exciting wavelength of 532 nm. Furthermore, the morphologies of the FLG samples were analyzed using TEM (JEOL TEM 2100) at 200 kV. HRTEM was employed to determine the microstructure characteristics of the FLG samples. The porous properties of the FLG samples were investigated using a Micromeritics TriStar II 3020 at a temperature of −273 °C. The pore size distributions of the samples were derived from the N_2_ adsorption–desorption isotherm, which was assessed using the Barrett–Joyner–Halenda (BJH) model. The specific surface areas (SSA) were evaluated using the Brunauer–Emmett–Teller (BET) method with a relative pressure (P/P_o_) of 0.001–1. Moreover, X-ray photoelectron spectroscopy (XPS) was employed to determine the primary compositions of the FLG samples.

### 2.4. Electrochemical Measurements

To measure the electrochemical performances of the FLG composites, the electrodes were assembled into a coin cell using a weight ratio (8:1:1) of active material, super P and polyvinylidene difluoride (PVDF) in *N*-methyl pyrrolidinone (NMP), respectively. This slurry was stirred vigorously overnight at room temperature and then homogenously applied onto a copper foil and dried overnight in a vacuum oven at a temperature of 110 °C. The cell was assembled in an Ar-filled glovebox with lithium foil as a cathode and a glass filter as separators. The electrolyte of LiPF6 (1.0 M) solution dissolved a volume ratio (1:1) of ethylene carbonate (EC) and dimethyl carbonate (DMC), which was used as an electrolyte. The galvanostatic charge–discharge (GCD) and specific capacity of the cell were determined using charging/discharging in a voltage range of 0.02–2.5 V (versus Li/Li^+^) at the ambient temperature under several current densities (Perkin Elmer VMP potentiostat/galvanostat). Furthermore, the cyclic voltammetry (CV) values were measured at a scan rate of 0.1 mVs^−1^ from 0.01 to 2.5 V.

## 3. Results and Discussion

### 3.1. Physicochemical Properties of the Composite FLG Materials

**Figure 1:**
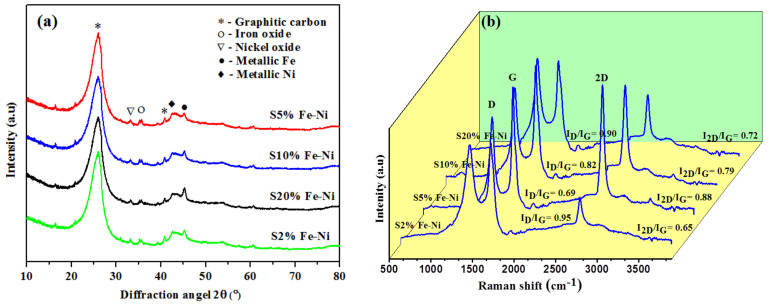
Results of: (**a**) XRD patterns and (**b**) Raman spectra for the different percentages of the double catalyst (Fe-Ni).

The typical XRD diffraction peaks of the porous carbon materials were catalytically graphitized at 1300 °C with different catalyst percentages (Figure 1). After graphitization for 20 min, the XRD patterns demonstrated two distinct peaks at 26° and 42.5°, which are related to the aromatic layers reflection plane of (002) and (100), respectively, and matched with the natural graphite [37]. XRD profiles also specify the microcrystalline structure of bituminous coal. The broad diffraction peak at 26° in the XRD profiles was observed in all Fe-Ni samples, from S2% to S20%, and the S5% sample had the highest intensify peak (see Figure 1a). Moreover, the synthetic graphite material had a lower intense peak at 45.2° for the Fe catalyst and 44.6° for the Ni catalyst. These results confirm that the Fe and Ni catalysts have existed in coal-based activated carbons, which is supported by the literature review [38]. The metallic Fe and Ni formed by reducing the iron oxides and nickel oxides during the graphitization period. Furthermore, the oxides and carbides of iron and nickel and the metallic Fe and Ni in the graphitized samples are beneficial for LIB applications. These oxides, such as Fe_2_O_3_ and NiO, are involved in the conversion reaction and delivered the extra sites to store the Li^+^ during the charging/discharging process. The metallic Fe and Ni assisted in raising the electronic conductivity [39,40,41].

The crystalline structures of the graphitic samples were further analyzed using several valuable parameters: grain size (La), thickness (Lc), interlayer distance (d_002_), degree of graphitization (I_D_/I_G_), and g factor. These values were calculated from the XRD and Raman data by employing the Scherrer and Bragg equation. In addition, the g factor was calculated using the Marie and Meiring equation [36] (Table 1).

The microcrystalline structures of all samples were further analyzed using the parameters of La, Lc, and d_002_, and the values are listed in Table 1. The crystal sizes and thicknesses of the double catalyst-loaded graphitized samples increased from the S2% to the S5% samples. The results of the S10% to S20% samples showed that their sizes and thicknesses were reduced due to the amount of catalytic agglomeration of the ordered structures. The particle size and thickness of the S5% sample were the highest of all of the samples, i.e., 2.30 and 4.95 nm, respectively, which indicated that the graphitic crystal stacking height and axial stacking increased. The composite materials’ graphitization value (g factor) was enriched dramatically, from 83.4% to 97.9%, increasing the catalyst percentages. However, after the S5% sample, the g factor values decreased due to the agglomeration effect. Moreover, the degree of graphitization (I_D_/I_G_) values decreased from the S2% to S5% samples, while the catalyst percentage increased, which occurred due to the catalytic agglomeration effect. The S5% sample demonstrated a low graphitization value of 0.69, indicating a high level of graphitic-ordered structures correlated with the XRD data. In addition, the interlayer spacing (d_002_) values declined as the catalyst percentage increased (see Table 1). The results showed that the lowest d_002_ value was for the S5% sample, which was 0.3355 nm, close to natural graphite (0.3354 nm), indicating that the crystal structure changed from amorphous to a graphitic network.

Moreover, the degree of the graphitization value was linked with the interlayer distance of the crystals. It has been shown that the microwave-assisted catalytic graphitization is more favorable than graphitization for making the shorter interlayer spacing in an electric furnace [42]. The La, Lc, and I_D_/I_G_ values demonstrated that the S5% sample exhibited a perfect crystal microstructure with the highest graphitization (g) value, i.e., 97.9% (Table 1).

The Raman spectra results (see Figure 1b) showed three distinct, prominent D, G, and 2D bands to confirm the graphitic microstructures further. The D band, at around 1350 cm^−1^, corresponded to the disordered carbon structures. The peaks at about 1580 and 2700 cm^−1^, denoted as the G and 2D bands, were related to highly ordered and regular graphitic microstructures [16,43]. Moreover, the distinct 2D band indicated that the analyzed samples contained FLG structures [16]. The graphitic ratio was measured from the intensity ratios of the D band and G band, which represented the degree of graphitization. The I_D_/I_G_ values decreased as the catalyst loading percentage increased from S2% to S5%, signifying the development of graphitic structures (see Figure 1b). The I_D_/I_G_ value for the S20% sample was 0.90, which was the highest due to the formation of more disordered carbon structures, and this correlated with the XRD data (see Table 1). In addition, Rodriguez et al. found that highly ordered carbon structures are favorable for high-performance anode materials in LIB applications, as they assisted in the intercalation of the lithium-ions in the carbon matrix [44].

Furthermore, the 2D peak (2700 cm^−1^) was a double resonance second-order overtone of the D peak and represented the number of graphene layers based on the location [45]. All of the catalyst loadings had the 2D band, which indicated a graphitic layer lattice in a carbon matrix. The I_2D_/I_G_ intensity ratio represented the number of the graphene layers. The results showed that the S5% sample had the highest value (I_2D_/I_G_ = 0.88) among all the samples, supporting the view that it had a multilayer structure. Moreover, the overall crystallinity of all samples was calculated by the I_2D_/I_D_ ratio, which is presented in Table 1. The results showed that the highest I_2D_/I_D_ value came from the S5% sample (1.27), which indicated an extended network structure [46,47].

**Figure 2:**
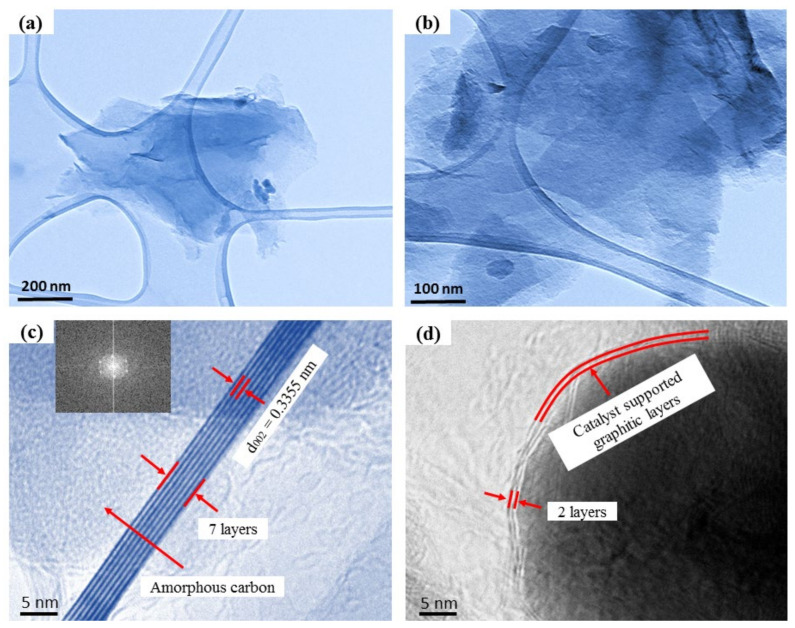
(**a**,**b**) Different magnifications of the TEM images of the S5% Fe-Ni sample; and (**c**,**d**) HRTEM images of the S5% Fe-Ni sample containing two to seven layers of graphene. The inset in (**c**) shows the FFT image of the corresponding HRTEM.

TEM and HRTEM were the most effective techniques further to confirm the crystallinity and structures of the graphitized samples. The micrograph for the S5% sample showed the transparent FLG nanosheets on a lacy carbon grid at several magnifications (Figure 2a,b). The catalytic graphitization of disordered carbon was a continuous driving process, including the precipitation of the amorphous carbon on the surfaces of the catalyst due to their ionization abilities and d-electron configuration. These properties assisted in precipitating the disordered carbon structures over the catalysts at the supersaturation point and growing the carbon layers through a dissolution and precipitation mechanism [48,49]. The metastable ferric carbide also formed an intermediate product from converting metallic oxides to Fe and Ni nanoparticles during the graphitization process. These metals acted as nuclei to make the graphitic carbon layers [39,50]. The phase diagram for the Fe and Ni demonstrated that the catalysts assisted in reducing the melting temperatures. As a result, the amorphous carbon dissolved on the catalysts to make the graphitic layers [34,35,49]. In addition, Fe played a role as a catalyst in reducing the graphitization temperature and informing the onion-like graphitic layer during the carbonization period [51]. Moreover, the well-developed Ni nanoparticles can produce ultrathin graphitic structures through the developed carbon structures and the amorphous carbon matrix. The carbon texture changed as the heating temperature increased, and the metallic Ni acted as nuclei to grow the graphitic layers [52]. Furthermore, the results showed that the fast heating rate during the graphitization period assisted in making the few-layer graphitic structures; nevertheless, a thick layer of graphitic carbon formed at lower heating temperatures [53].

The HRTEM image of the S5% sample reveals nanosheets consisting of around two to seven layers of graphene sheets, as shown in Figure 2c,d. In addition, the HRTEM image in Figure 2c shows a few layers of the sheets over a sheet distance of around 0.3355 nm, which resembled the (002) plane of the FLG nanosheets. The HRTEM image of the S5% sample clarifies that the graphene-based material had a highly crystalline structure. The correlating fast Fourier transform (FFT) image (inset in Figure 2c) demonstrated the six-fold symmetry of the graphene [54]. Furthermore, it also showed the crystalline nature of the FLG composite material.

**Figure 3:**
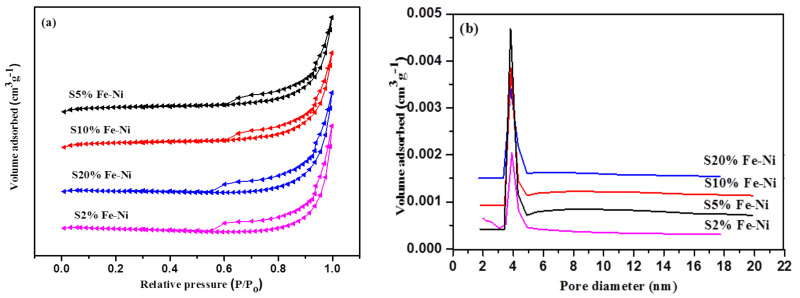
(**a**) N_2_ adsorption–desorption isotherms; and (**b**) Pore-size distribution curves for the different percentages of the Fe-Ni catalyst.

The N_2_ adsorption–desorption isotherm determined the physical structures of the FLG materials (see Figure 3a). The specific surface area of the S5% sample was calculated to be 175.61 m^2^g^−1^, which was the highest among all of the samples (see Table 1). The S2% sample contained the lowest surface area (81.92 m^2^g^−1^), which was also higher than natural graphite (5.5 m^2^g^−1^) [36] and anthracite coal-based materials (4.7–6.8 m^2^g^−1^) [17]. In addition, the FLG samples demonstrated a Type IV isotherm hysteresis loop, which indicated a wide range of particle size distributions (2–50 nm) representing the mesopores structure [55]. The density functional theory calculated the pore sizes of the FLG samples, and the S5% sample showed the largest particle size at the peak of around 4.3 nm (see Figure 3b).

**Figure 4:**
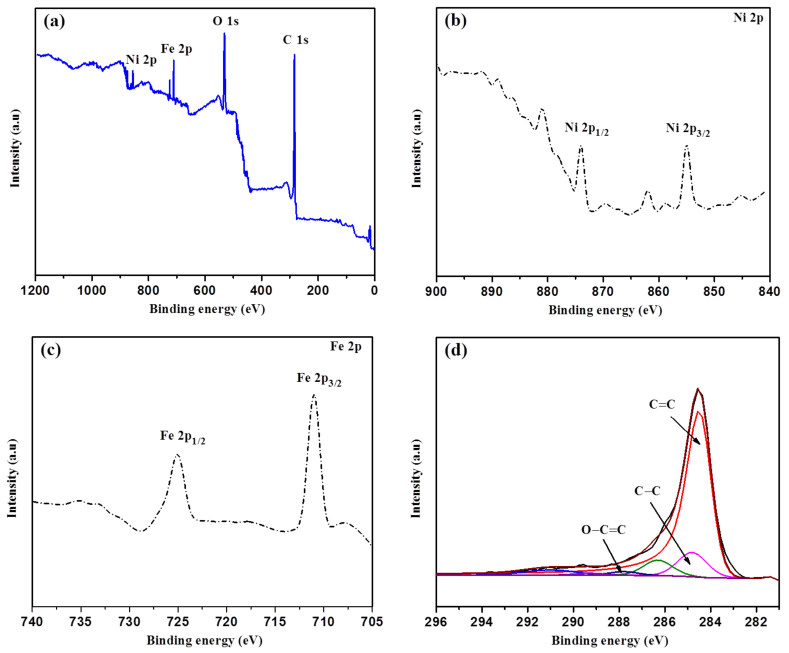
XPS analysis of the S5% Fe-Ni sample: (**a**) Survey scan; (**b**) Ni 2p spectra; (**c**) Fe 2p spectra; and (**d**) Deconvoluted XPS spectra of the C 1s region.

The surface chemical composition of the S5% sample was investigated using XPS (Figure 4). The survey spectrum exposed the existence of the Fe, Ni, C, N, and O elements at 710.0, 854.1, 283.99, and 530.4 eV (see Figure 4a). In addition, the elemental composition details examined through a high-resolution spectrum and the sharp peak spectrum at 854.5 and 873.7 eV in the Ni 2p region corresponded to Ni 2p_3/2_ and Ni 2p_1/2_, respectively (see Figure 4b) [56]. The peaks of Fe 2p at around 710.8 eV and 724.9 eV suggested the peaks of Fe 2p_3/2_ and Fe 2p_1/2_, respectively (see Figure 4c), which indicated that the Fe 2p was generally derived from the Fe^3+^ [57]. Both of the metallic elements (Fe and Ni) confirmed the deposition on the FLG surface, which was in line with the XRD data. Furthermore, the highly intensive spectrum of C 1s is shown in Figure 4d and is convoluted into three prominent peaks. The predominant peaks are centered at 284.5, 284.8, and 286.3 eV, which are ascribed to the C=C (sp^2^), C-C (sp^3^), and C-O bonds, respectively [58,59]. Moreover, the O 1s spectrum showed the C=O bond at 287.8 eV and the O-C=O bond position at 289.3 eV. These properties (O_2_-containing functional groups and N-doping) have improved the storage capacity and affinity between the surfaces and ions for LIB applications [60]. Furthermore, the catalysts were decomposed into oxides and consequently reduced to metallic elements during the pyrolysis. The carbon and catalyst interactions, diffusion effect, and external forces play crucial roles in transforming the disordered carbon into graphitic nanostructures according to the dissolution–precipitation mechanism [61].

### 3.2. GCD and CV Analysis of the Composite FLG Materials

**Figure 5:**
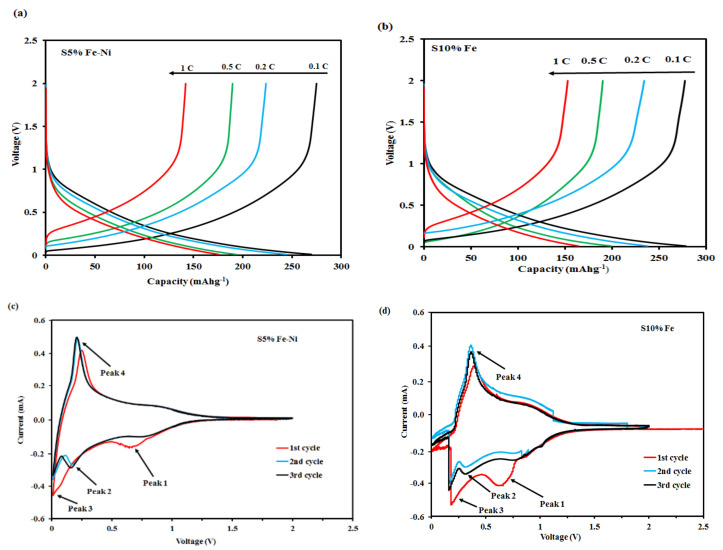
Electrochemical performances of the FLG materials: (**a**,**b**) GCD curves of the S5% Fe-Ni and S10% Fe samples at rates of 0.1 to 1 C; (**c**,**d**) CV curves of the S5% Fe-Ni and S10% Fe samples at a scan rate of 0.1 mVs^−1^.

The galvanostatic charge–discharge (GCD) curves for the single and double catalyst-loaded S5% Fe-Ni and S10% Fe samples at the current rates of between 0.1 and 1 C during the first cycle are shown in Figure 5a,b respectively. The first discharge cycle curve displays two voltage plateaus that correlate with a natural graphitic composite material [62]. The discharge voltage plateau at around 0.72 V corresponded with the formation of a solid electrolyte interphase film. However, the discharge voltage plateau at 0.2 V represents the extraction of lithium-ions from the structure of the graphite [63].

The discharge capacities of the S5% Fe-Ni and S10% Fe samples at the first cycle were 270.4 mAhg^−1^ and 255.25 mAhg^−1^, respectively, while the charge capacities were 251.6 mAhg^−1^ and 248.68 mAhg^−1^, respectively. In addition, at a high current rate (1 C), the charge capacity of the S5% Fe-Ni sample was 180.38 mAhg^−1^. However, the result for the S10% Fe sample was a lower charge capacity of 151.38 mAhg^−1^. The single and double catalysts results showed that the charge capacities reduced as the current density increased. Therefore, the dual catalyst demonstrated a more negligible polarization at a high current rate [7].

The cyclic voltammetry (CV) curves for the S5% Fe-Ni and S10% Fe samples were investigated at a scan rate of 0.1 mVs^−1^ with a voltage range from 0.01 to 2.5 V (see Figure 5c,d). In the first cycle, the CV curves for the S5% Fe-Ni and S10% Fe samples displayed the two well-defined and prominent reduction peaks (peak-1) at 0.5 V and 0.8 V, respectively. It corresponded to the formation of the solid electrolyte interphase (SEI) film on the surfaces of the samples. It also indicated the decomposition of the metallic oxides (Fe and Ni) into metallic (Fe and Ni) metal during the Li^+^ intercalation process [64]. The broad reduction peak is found at the first cycle, which proposes the solid electrolyte interface (SEI) film formation on the composite electrode surface. In addition, a cathodic peak observed between 0.5 and 0.8 V suggests reversible lithium insertion into the carbon layers and nanopores composite materials. Moreover, at the first cycle, the cathodic peak intensity is much higher than for the other cycles, which may happen due to the SEI formation, the decomposition of electrolyte, and the Li^+^ irreversible insertion into the carbon material’s particular locations [65,66,67]. Furthermore, it is noted that this peak disappeared in the second and third cycles of the S5% Fe-Ni sample, which was designated when the stable SEI films formed. However, peak-1 was also present in the S10% Fe sample results, indicating the instability of the SEI film. In the subsequent reduction process, a weak peak (peak-2) was observed at 0.21 V in the following cycle, suggesting the irreversible storage of Li^+^ in the composite material, which generated a stable SEI layer form [68,69]. In addition, peak-3 was at around 0 V for the S5% Fe-Ni sample, which demonstrated the intercalation of the lithium-ions into the graphitic structures of the synthetic materials. However, the position of the S10% Fe sample peak-3 was far from 0 V, and it did not show the intercalation of lithium ions into the samples [68]. A sharp oxidation peak (peak-4) at around 0.25 V connected with the Li^+^ de-intercalation process during the charging process [68]. Finally, the cyclic voltammetry curves of the S5% Fe-Ni sample overlapped in the second and third cycles, which signposted the excellent electrochemical stability of the FLG for anode materials [70]. Meanwhile, the curves for the single catalyst (S10% Fe) sample did not overlap on the second and third cycles, as shown in Figure 5d.

### 3.3. The Rate Capability, Cycling Stability, and Coulombic Efficiency of the Composite FLG Materials

**Figure 6:**
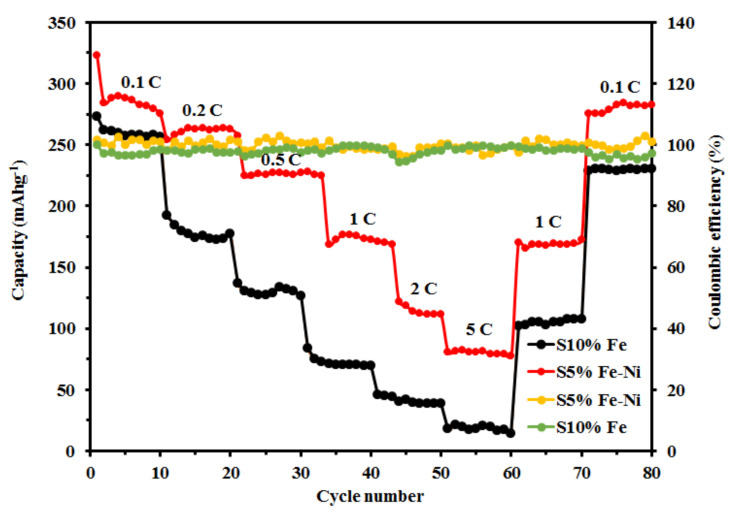
The rate capabilities and Coulombic efficiencies of the S5% Fe-Ni and S10% Fe samples.

The rate performances of the single S10% Fe and double S5% Fe-Ni catalyst samples at different current densities, from 0.1 to 5.0 C, over ten cycles for each sample are shown in Figure 6. The results for the S5% Fe-Ni sample showed high average reversible capacities for 10 cycles of 287.91 mAhg^−1^, 261.39 mAhg^−1^, 229.31 mAhg^−1^, 189.45 mAhg^−1^, 131.14 mAhg^−1^, and 80.37 mAhg^−1^, which were found at the current densities of 0.1 C, 0.2 C, 0.5 C, 1 C, 2 C, and 5 C, respectively. However, the S10% Fe sample showed a reversible capacity of 260.13 mAhg^−1^ at 0.1 C, 178.07 mAhg^−1^ at 0.2 C, 130.29 mAhg^−1^ at 0.5 C, 72.43 mAhg^−1^ at 1 C, 41.25 mAhg^−1^ at 2 C, and 18.29 mAhg^−1^ at 5 C, respectively. These results demonstrated that the dual S5% Fe-Ni catalyst contained a higher reversible capacity than the single S10% Fe catalyst at the current rate of 0.1 C. Furthermore, the S5% Fe-Ni sample returned the high capabilities of 168.89 mAhg^−1^ and 280.15 mAhg^−1^ after reverting to the current rates of 1 C and 0.1 C, respectively, which demonstrated the excellent stable electrochemical properties of the S5% Fe-Ni catalyst. These results are significantly higher than those reported for other 3D graphene materials (84 mAhg^−1^ and 184 mAhg^−1^) [71,72]. However, the single S10% Fe catalyst results showed reversible capacities of 105.23 mAhg^−1^ and 229.85 mAhg^−1^ at current densities of 1 C and 0.1 C, respectively, which are lower than the results for the double S5% Fe-Ni catalyst. The superior rate capability of the S5% Fe-Ni electrode material significantly contributed to the connections with the micro-, meso-, and macroporous structures and the functional groups. Porous structures such as micro-, meso-, and macropores act as functional groups activated by ion accommodation to achieve a high storage capacity, facilitate ion transport through nano-channels, and reduce distance Li-ion diffusion. Overall, the composite S5% Fe-Ni sample has ion lodging functional groups, ion transportation, and shorter distance in an inner surface and promoted more active sites with sufficient intercalation spaces.

The GCD test determines the Coulombic efficiencies of the S5% Fe-Ni and S10% Fe samples at a current rate of 0.1 C for eighty cycles (Figure 6). The dual S5% Fe-Ni catalyst sample results showed an average Coulombic efficiency of 99.9% during the cycling performance test. However, the Coulombic efficiency of the single S10% Fe catalyst sample was 97.96%. The Coulombic efficiency of the S5% Fe-Ni electrode material was in a stable range between 98% and 100% during the cycling. Therefore, the S5% Fe-Ni sample findings suggested that the catalyst’s efficiency improved with the graphitization values during the pyrolysis period and decreased the graphitization heating temperature while providing a highly ordered graphite structure, which was supported by the literature on coal-based catalytic graphitization [33]. Furthermore, a highly ordered layer structure could enable Li-ions and electron transfer migration and indicated a boost in the Li-ion storage capacity with excellent electrochemical performances.

**Figure 7:**
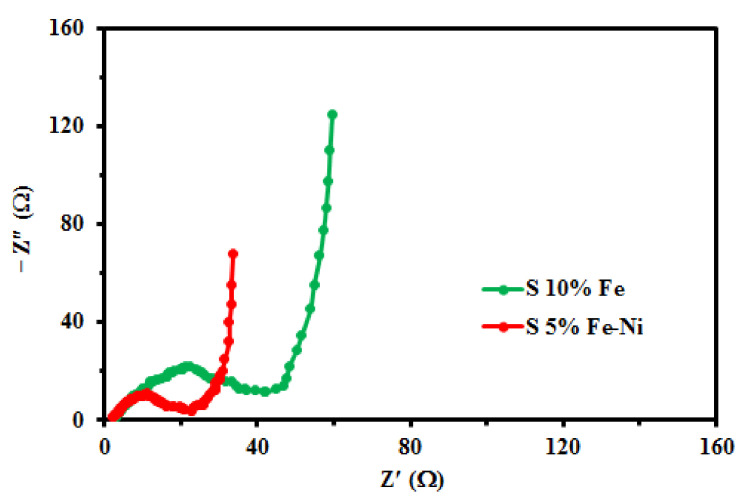
The Nyquist plots of the S5% Fe-Ni and S10% Fe samples.

The electrochemical impedance spectroscopy (EIS) experiments were carried out to understand further the electrochemical behavior and its potential application of the S5% Fe-Ni and S10% Fe composite samples. The semicircle EIS curves of S5% Fe-Ni were smaller than the S10% Fe, as shown in Figure 7. According to the literature survey, this type of equivalent circuit enlightened the anode impedance spectra [73]. At the high to medium-frequency region, the two composites are partially overlapped by semicircles. In addition, the charge transfer resistance of the S5% Fe-Ni and S10% Fe samples were 4 ohm and 12 ohm, which are compliant with the CV result. The electronic conductivity of the S5% Fe-Ni sample represented a crucial role in charge transfer for batteries. It increased to make the short distance of lithium-ion move during the cycling period.

**Figure 8:**
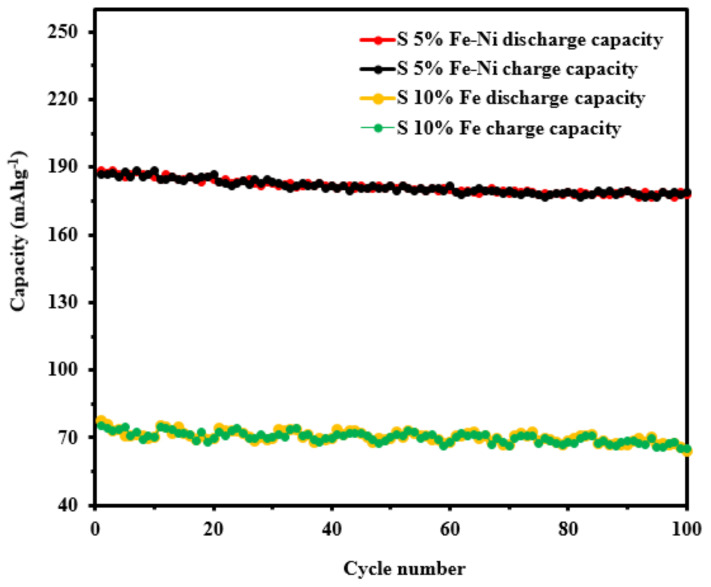
Cycling stability of the S5% Fe-Ni and S10% Fe samples at the current rate of 1 C.

The cycling performance of anode material is one of the most vital parameters for LIBs application. The cycling performance of the S5% Fe-Ni sample was conducted by the GCD test repeating at a 1C current rate for 100 cycles, as shown in Figure 8. The initial capacity of the S5% Fe-Ni sample was decayed by the solid electrolyte interphase (SEI) formation with the lithium-ion reduction and increased the inner electrode. In contrast, the dual catalyst of S5% Fe-Ni retained a higher capacity than the single catalyst (S10% Fe). The initial capacity of the S5% Fe-Ni was 187 mAhg^−1^ after 10 cycles. In contrast, a single catalyst (S10% Fe) capacity was 72.33 mAhg^−1^ at the same cycles. After 100 cycles, the single catalyst (S10% Fe) capacity was 67.5 mAhg^−1^ and the dual catalyst was 177.78 mAhg^−1^, respectively. In addition, the corresponding capacity reached as high as 95% after 100 cycles of the dual catalyst-loaded sample. The structural change of the sample significantly improves by the dual catalyst the LIBs’ performance.

## 4. Conclusions

Coal-based FLG materials were successfully fabricated from bituminous coal through dual catalytic graphitization (Fe and Ni) at 1300 °C under the microwave process. The Raman spectra of the synthesized FLGs exhibited a 2D band feature at 2700 cm^−1^. A well-developed graphitic structure with two to seven layers was achieved using the S5% Fe-Ni catalyst and required only 20 min using a microwave. The analysis indicated that uniform and constant graphene sheets were formed. The results showed that the growth of the FLG using a dissolution–precipitation mechanism mainly depended on the catalyst-loading percentage and the MW heating temperature. The prepared FLG possessed a highly ordered graphitic layer, which was favorable for the rapid transportation of Li-ions.

Moreover, the S5% Fe-Ni catalyst exhibited a high reversible capacity of 287.91 mAhg^−1^ at 0.1 C, which was still 280.15 mAhg^−1^ after eighty cycling performances and had a high Coulombic efficiency of 99.9%. However, the single S10% Fe catalyst had a reversible capacity of 260.13 mAhg^−1^ at 0.1 C, with a Coulombic efficiency of 97.96%. The dual S5% Fe-Ni catalyst-loaded FLG sample results indicated that the composite material had excellent cycling stability. After 100 cycling performances, the dual catalyst (S5% Fe-Ni) capacity was 187 mAhg^−1^, and the single catalyst capacity (S10% Fe) was 67.5 mAhg^−1^. Furthermore, the dual catalyst-loaded sample retention capacity was 95% after 100 cycles. These unique characteristics of the FLG significantly supported the findings that stable SEI formed, the conductivity improved, and the ion diffusion path was efficiently shortened during the cycling process.

The elemental composition analysis represented that the dual catalyst was successfully obtained in the composite material. In addition, the EIS and cyclic voltammetry results for the prepared dual catalyst (S5% Fe-Ni)-loaded FLG materials revealed excellent stable electrochemical properties. Considering the plentiful reserves, cheap precursor, and superior electrochemical performances of the prepared FLG materials suggests an effective strategy for commercializing coal-based graphite for applications as an anode material in LIBs.

## Figures and Tables

**Table 1:** Structural properties for the different percentages of the double catalyst (Fe-Ni).

Fe–Ni Loading (%)/Parameters	S2	S5	S10	S20
Interlayer spacing (d_002_) (nm) ^1^	0.3368	0.3355	0.3361	0.3366
Crystallite size (Lc) (nm) ^1^	2.01	2.30	2.12	2.07
Crystallite thickness (La) (nm) ^1^	4.34	4.95	4.57	4.45
I_D_/I_G_	0.95	0.69	0.82	0.90
I_2D_/I_G_	0.65	0.88	0.79	0.72
I_2D_/I_D_	0.68	1.27	0.96	0.80
Surface area (m^2^g^−1^)	81.92	175.61	136.23	97.47
“g” factor (%) ^2^	83.4	97.9	92.3	86.4

^1^ Corresponds to the (002) graphitic plane from the XRD data. ^2^ Degree of graphitization according to the Marie and Meiring equation, g = (0.344–d002)/(0.344–0.3354).

## Data Availability

Data contained within this article.

## References

[1] Xu J., Dou Y., Wei Z., Ma J., Deng Y., Li Y., Liu H.K., Dou S.X. (2017). Recent Progress in Graphite Intercalation Compounds for Rechargeable Metal (Li, Na, K, Al)-Ion Batteries. Adv. Sci..

[2] Cheng X.-B., Zhang R., Zhao C.-Z., Zhang Q. (2017). Toward Safe Lithium Metal Anode in Rechargeable Batteries: A Review. Chem. Rev..

[3] Etacheri V., Marom R., Elazari R., Salitra G., Aurbach D. (2011). Challenges in the development of advanced Li-ion batteries: A review. Energy Environ. Sci..

[4] Marom R., Amalraj S.F., Leifer N., Jacob D., Aurbach D. (2011). A review of advanced and practical lithium battery materials. J. Mater. Chem..

[5] Luo B., Zhi L. (2015). Design and construction of three dimensional graphene-based composites for lithium ion battery applications. Energy Environ. Sci..

[6] Zhang J., Cao H., Tang X., Fan W., Peng G., Qu M. (2013). Graphite/graphene oxide composite as high capacity and binder-free anode material for lithium ion batteries. J. Power Sources.

[7] Huang S., Guo H., Li X., Wang Z., Gan L., Wang J., Xiao W. (2013). Carbonization and graphitization of pitch applied for anode materials of high power lithium ion batteries. J. Solid State Electrochem..

[8] Wang Z., Zhang X., Liu X., Zhang Y., Zhao W., Li Y., Qin C., Bakenov Z. (2020). High specific surface area bimodal porous carbon derived from biomass reed flowers for high performance lithium-sulfur batteries. J. Colloid Interface Sci..

[9] Kim S.Y., Song Y.I., Wee J.-H., Kim C.H., Ahn B.W., Lee J.W., Shu S.J., Terrones M., Kim Y.A., Yang C.-M. (2019). Few-layer graphene coated current collectors for safe and powerful lithium ion batteries. Carbon.

[10] Delhaes P. (2000). Graphite and Precursors.

[11] Fan C.-L., He H., Zhang K.-H., Han S.-C. (2012). Structural developments of artificial graphite scraps in further graphitization and its relationships with discharge capacity. Electrochim. Acta.

[12] Mathur R.B., Lal C., Sharma D.K. (2007). Catalyst-Free Carbon Nanotubes from Coal-Based Material. Energy Sources Part A Recover. Util. Environ. Eff..

[13] Du A.-B., Liu X.-G., Fu D.-J., Han P.-D., Xu B.-S. (2007). Onion-like fullerenes synthesis from coal. Fuel.

[14] Sasikala S.P., Henry L., Tonga G.Y., Huang K., Das R., Giroire B., Marre S., Rotello V.M., Penicaud A., Poulin P. (2016). High Yield Synthesis of Aspect Ratio Controlled Graphenic Materials from Anthracite Coal in Supercritical Fluids. ACS Nano.

[15] Haenel M.W. (1992). Recent progress in coal structure research. Fuel.

[16] Xing B., Zhang C., Cao Y., Huang G., Liu Q., Zhang C., Chen Z., Yi G., Chen L., Yu J. (2018). Preparation of synthetic graphite from bituminous coal as anode materials for high performance lithium-ion batteries. Fuel Process. Technol..

[17] Cameán I., LaVela P., Tirado J.L., Garcia A. (2010). On the electrochemical performance of anthracite-based graphite materials as anodes in lithium-ion batteries. Fuel.

[18] Tian Z.Q., Jiang S.P., Liang Y.M., Shen P.K. (2006). Synthesis and Characterization of Platinum Catalysts on Multiwalled Carbon Nanotubes by Intermittent Microwave Irradiation for Fuel Cell Applications. J. Phys. Chem. B.

[19] Liu X., Quan X., Bo L., Chen S., Zhao Y., Chang M. (2004). Temperature measurement of GAC and decomposition of PCP loaded on GAC and GAC-supported copper catalyst in microwave irradiation. Appl. Catal. A Gen..

[20] Foo K.Y., Hameed B.H. (2011). Preparation of oil palm (Elaeis) empty fruit bunch activated carbon by microwave-assisted KOH activation for the adsorption of methylene blue. Desalination.

[21] Liu Q.-S., Zheng T., Li N., Wang P., Abulikemu G. (2010). Modification of bamboo-based activated carbon using microwave radiation and its effects on the adsorption of methylene blue. Appl. Surf. Sci..

[22] Zhu Y., Murali S., Stoller M.D., Velamakanni A., Piner R.D., Ruoff R.S. (2010). Microwave assisted exfoliation and reduction of graphite oxide for ultracapacitors. Carbon.

[23] Yuan S.-J., Dong B., Dai X.-H. (2021). Facile and scalable synthesis of high-quality few-layer graphene from biomass by a universal solvent-free approach. Appl. Surf. Sci..

[24] Cai W., Zhu Y., Li X., Piner R.D., Ruoff R.S. (2009). Large area few-layer graphene/graphite films as transparent thin conducting electrodes. Appl. Phys. Lett..

[25] Islam F., Tahmasebi A., Wang R., Yu J. (2021). Structure of Coal-Derived Metal-Supported Few-Layer Graphene Composite Materials Synthesized Using a Microwave-Assisted Catalytic Graphitization Process. Nanomaterials.

[26] Marsh H., Crawford D., Taylor D. (1983). Catalytic graphitization by iron of isotropic carbon from polyfurfuryl alcohol, 725–1090 K. A high resolution electron microscope study. Carbon.

[27] Maldonado-Hódar F.J., Moreno-Castilla C., Rivera-Utrilla J., Hanzawa Y., Yamada Y. (2000). Catalytic Graphitization of Carbon Aerogels by Transition Metals. Langmuir.

[28] Gutierrez-Pardo A., Ramírez-Rico J., De Arellano-López A.R., Martínez-Fernández J. (2014). Characterization of porous graphitic monoliths from pyrolyzed wood. J. Mater. Sci..

[29] Li S., Li F., Wang J., Tian L., Zhang H., Zhang S. (2018). Preparation of Hierarchically Porous Graphitic Carbon Spheres and Their Applications in Supercapacitors and Dye Adsorption. Nanomaterials.

[30] Tang D.-M., Liu C., Yu W.-J., Zhang L.-L., Hou P.-X., Li J.-C., Li F., Bando Y., Golberg D., Cheng H.-M. (2014). Structural Changes in Iron Oxide and Gold Catalysts during Nucleation of Carbon Nanotubes Studied by In Situ Transmission Electron Microscopy. ACS Nano.

[31] Yu Z.-L., Xin S., You Y., Yu L., Lin Y., Xu D.-W., Qiao C., Huang Z.-H., Yang N., Yu S.-H. (2016). Ion-Catalyzed Synthesis of Microporous Hard Carbon Embedded with Expanded Nanographite for Enhanced Lithium/Sodium Storage. J. Am. Chem. Soc..

[32] Arrigo R., Sasaki T., Callison J., Gianolio D., Schuster M.E. (2022). Monitoring dynamics of defects and single Fe atoms in N-functionalized few-layer graphene by in situ temperature programmed scanning transmission electron microscopy. J. Energy Chem..

[33] Wang T., Wang Y., Cheng G., Ma C., Liu X., Wang J., Qiao W., Ling L. (2020). Catalytic Graphitization of Anthracite as an Anode for Lithium-Ion Batteries. Energy Fuels.

[34] Bystrzejewski M. (2011). Synthesis of carbon-encapsulated iron nanoparticles via solid state reduction of iron oxide nanoparticles. J. Solid State Chem..

[35] Ohtani H., Hasebe M., Nishizawa T. (1984). Calculation of Fe-C, Co-C and Ni-C phase diagrams. Trans. Iron Steel Inst. Jpn..

[36] Yeh T.-S., Wu Y.-S., Lee Y.-H. (2011). Graphitization of unburned carbon from oil-fired fly ash applied for anode materials of high power lithium ion batteries. Mater. Chem. Phys..

[37] Badenhorst H. (2014). Microstructure of natural graphite flakes revealed by oxidation: Limitations of XRD and Raman techniques for crystallinity estimates. Carbon.

[38] Gao S., Tang Y., Wang L., Liu L., Jia D., Zhao Z. (2017). NiFe nanoalloys in-situ immobilized on coal based activated carbons through one-step pyrolysis as magnetically recoverable catalysts for reduction of 4-nitrophenol. J. Alloys Compd..

[39] Li J., Wen W., Xu G., Zou M., Huang Z., Guan L. (2015). Fe-added Fe3C carbon nanofibers as anode for Li ion batteries with excellent low-temperature performance. Electrochim. Acta.

[40] Zhang J., Tahmasebi A., Omoriyekomwan J.E., Yu J. (2021). Microwave-assisted synthesis of biochar-carbon-nanotube-NiO composite as high-performance anode materials for lithium-ion batteries. Fuel Process. Technol..

[41] Zhang L., Bin Wu H., Lou X.W.D. (2014). Iron-Oxide-Based Advanced Anode Materials for Lithium-Ion Batteries. Adv. Energy Mater..

[42] Kim T., Lee J., Lee K.-H. (2016). Full graphitization of amorphous carbon by microwave heating. RSC Adv..

[43] Tang J., Salunkhe R., Zhang H., Malgras V., Ahamad T., AlShehri S.M., Kobayashi N., Tominaka S., Ide Y., Kim J.H. (2016). Bimetallic Metal-Organic Frameworks for Controlled Catalytic Graphitization of Nanoporous Carbons. Sci. Rep..

[44] Rodriguez E., Cameán I., García R., Garcia A. (2011). Graphitized boron-doped carbon foams: Performance as anodes in lithium-ion batteries. Electrochim. Acta.

[45] Robertson A.W., Warner J.H. (2011). Hexagonal Single Crystal Domains of Few-Layer Graphene on Copper Foils. Nano Lett..

[46] Sharma B., Schumann T., de Oliveira M.H., Lopes J.M.J. (2017). Controlled synthesis and characterization of multilayer graphene films on the C-face of silicon carbide. Phys. Status Solidi.

[47] Santangelo S., Messina G., Faggio G., Lanza M., Milone C. (2011). Evaluation of crystalline perfection degree of multi-walled carbon nanotubes: Correlations between thermal kinetic analysis and micro-Raman spectroscopy. J. Raman Spectrosc..

[48] Ōya A., Ōtani S. (1979). Catalytic graphitization of carbons by various metals. Carbon.

[49] Derbyshire F., Presland A., Trimm D. (1975). Graphite formation by the dissolution—Precipitation of carbon in cobalt, nickel and iron. Carbon.

[50] Xiong W., Zhou Y.S., Hou W.J., Guillemet T., Silvain J.F., Gao Y., Lahaye M., Lebraud E., Xu S., Wang X.W. (2015). Solid-state graphene formation via a nickel carbide intermediate phase. RSC Adv..

[51] Zhao M., Song H., Chen X., Lian W. (2007). Large-scale synthesis of onion-like carbon nanoparticles by carbonization of phenolic resin. Acta Mater..

[52] Liu Y., Liu Q., Gu J., Kang D., Zhou F., Zhang W., Wu Y., Zhang D. (2013). Highly porous graphitic materials prepared by catalytic graphitization. Carbon.

[53] Juang Z.-Y., Wu C.-Y., Lo C.-W., Chen W.-Y., Huang C.-F., Hwang J.-C., Chen F.-R., Leou K.-C., Tsai C.-H. (2009). Synthesis of graphene on silicon carbide substrates at low temperature. Carbon.

[54] Zan R. (2013). Microscopy and Spectroscopy of Graphene: Atomic Scale Structure and Interaction with Foreign Atom Species.

[55] Sekar S., Lee Y., Kim D.Y., Lee S. (2019). Substantial LIB Anode Performance of Graphitic Carbon Nanoflakes Derived from Biomass Green-Tea Waste. Nanomaterials.

[56] Tan Z., Zhang W., Qian D., Cui C., Xu Q., Li L., Li S., Li Y. (2012). Solution-processed nickel acetate as hole collection layer for polymer solar cells. Phys. Chem. Chem. Phys..

[57] Graat P.C., Somers M.A.J. (1996). Simultaneous determination of composition and thickness of thin iron-oxide films from XPS Fe 2p spectra. Appl. Surf. Sci..

[58] Varghese B., Reddy M.V., Yanwu Z., Lit C.S., Hoong T.C., Rao G.V.S., Chowdari B.V.R., Wee A., Lim C.T., Sow C.-H. (2008). Fabrication of NiO Nanowall Electrodes for High Performance Lithium Ion Battery. Chem. Mater..

[59] Yue H., Shi Z., Wang Q., Cao Z., Dong H., Qiao Y., Yin Y., Yang S. (2014). MOF-derived cobalt-doped ZnO@ C composites as a high-performance anode material for lithium-ion batteries. ACS Appl. Mater. Interfaces.

[60] Wang L., Zhuo L., Cheng H., Zhang C., Zhao F. (2015). Porous carbon nanotubes decorated with nanosized cobalt ferrite as anode materials for high-performance lithium-ion batteries. J. Power Sources.

[61] Wang K., Cao Y., Wang X., Kharel P.R., Gibbons W., Luo B., Gu Z., Fan Q., Metzger L. (2016). Nickel catalytic graphitized porous carbon as electrode material for high performance supercapacitors. Energy.

[62] Wu X., Yang X., Zhang F., Cai L., Zhang L., Wen Z. (2017). Carbon-coated isotropic natural graphite spheres as anode material for lithium-ion batteries. Ceram. Int..

[63] Liu T., Luo R., Qiao W., Yoon S.-H., Mochida I. (2010). Microstructure of carbon derived from mangrove charcoal and its application in Li-ion batteries. Electrochim. Acta.

[64] Zhu C., Akiyama T. (2016). Cotton derived porous carbon via an MgO template method for high performance lithium ion battery anodes. Green Chem..

[65] Liu L., Yang L., Wang P., Wang C.-Y., Cheng J., Zhang G., Gu J.-J., Cao F.-F. (2017). Porous nitrogen-doped carbon derived from peanut shell as anode material for lithium ion battery. Int. J. Electrochem. Sci..

[66] Gao F., Geng C., Xiao N., Qu J., Qiu J. (2018). Hierarchical porous carbon sheets derived from biomass containing an activation agent and in-built template for lithium ion batteries. Carbon.

[67] Fan W., Zhang H., Wang H., Zhao X., Sun S., Shi J., Huang M., Liu W., Zheng Y., Li P. (2019). Dual-doped hierarchical porous carbon derived from biomass for advanced supercapacitors and lithium ion batteries. RSC Adv..

[68] Ru H., Xiang K., Zhou W., Zhu Y., Zhao X.S., Chen H. (2016). Bean-dreg-derived carbon materials used as superior anode material for lithium-ion batteries. Electrochim. Acta.

[69] Naji A., Ghanbaja J., Willmann P., Billaud D. (1997). Electrochemical reduction of graphite in LiClO4-propylene carbonate electrolyte: Influence of the nature of the surface protective layer. Carbon.

[70] Yue X., Sun W., Zhang J., Wang F., Yang Y., Lu C., Wang Z., Rooney D., Sun K. (2016). Macro-mesoporous hollow carbon spheres as anodes for lithium-ion batteries with high rate capability and excellent cycling performance. J. Power Sources.

[71] Jiang Y., Jiang Z.-J., Cheng S., Liu M. (2014). Fabrication of 3-Dimensional Porous Graphene Materials for Lithium Ion Batteries. Electrochim. Acta.

[72] Ramos A., Cameán I., Cuesta N., García A.B. (2014). Graphitized stacked-cup carbon nanofibers as anode materials for lithium-ion batteries. Electrochim. Acta.

[73] Kim C., Jung J.-W., Yoon K.R., Youn D.-Y., Park S., Kim I.-D. (2016). A High-Capacity and Long-Cycle-Life Lithium-Ion Battery Anode Architecture: Silver Nanoparticle-Decorated SnO2/NiO Nanotubes. ACS Nano.

